# Photothermal imaging of cellular responses to glucose deprivation†

**DOI:** 10.1039/d4cb00269e

**Published:** 2025-01-31

**Authors:** Jun Miyazaki, Ryotaro Wagatsuma, Koji Okamoto

**Affiliations:** a Faculty of Systems Engineering, Wakayama University Wakayama 640-8510 Japan jmiya@wakayama-u.ac.jp; b Graduate School of Frontier Biosciences, Osaka University Osaka 565-0871 Japan

## Abstract

In solid tumours, cancer cells modify their metabolic processes to endure environments with nutrient and oxygen scarcity due to inadequate blood flow. A thorough understanding of this adaptive mechanism, which requires reliable microscopic techniques, is crucial for developing effective cancer treatments. In the present study, we used multi-wavelength photothermal (PT) microscopy to visualise the cellular response to glucose deprivation in living cells derived from cervical cancer. We found increased mitochondrial PT signal intensity under glucose deprivation conditions, which is indicative of a correlation between mitochondrial crista density and PT signal intensity. Furthermore, PT microscopy revealed that the activity of the autophagy-lysosome system can be evaluated by detecting substances accumulated in lysosomes. Using this method, we confirmed that ferritin and denatured proteins from the endoplasmic reticulum were present within the lysosomes. The detectability of these substances using PT microscopy at visible wavelengths indicated the presence of iron ions. This method does not require labeling of molecules and provides reliable information and detailed insights into the cellular responses associated with the adaptation of cancer cell metabolism to nutrient stress conditions.

## Abstract 2

Cellular responses to glucose deprivation in live cancer cells were visualised using multi-wavelength photothermal microscopy without the use of labelling molecules.

## Graphical abstract

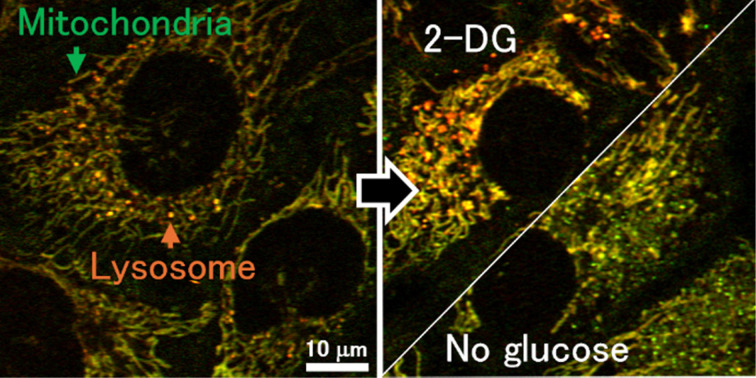

## Introduction

The rapid proliferation of cancer cells requires substantial nutrient uptake. When tumour expansion outpaces neovascularisation, the cancer cells are faced with nutrient and oxygen deprivation. Cancer cells are particularly dependent on glycolysis for production of adenosine triphosphate (ATP), and are therefore, prone to glucose deprivation.^1^ Glucose deprivation results in ATP depletion, increase in reactive oxygen species,^2–4^ and endoplasmic reticulum (ER) stress^5^ because of the accumulation of unfolded or misfolded proteins. Whereas normal cells generally die under these conditions, cancer cells adapt their metabolism for survival. For example, they enhance oxidative phosphorylation in mitochondria for ATP production when glucose is scarce.^6^ The unfolded protein response is highly activated in many types of cancers, which ensures maintenance of protein homeostasis in the ER.^7,8^ Furthermore, autophagy, a major intracellular degradation system involving lysosomes, is activated in cancer cells to eliminate misfolded proteins and damaged organelles.^3,9^ Recent findings suggest that cancer cells with activated stress responses become resistant to various anticancer drugs, consequently promoting disease progression.^10–12^ Considering the variety of adaptive mechanisms employed by cancer cells, extensive research has been focused on understanding the regulatory mechanisms of stress responses in cancer and developing drugs that target these responses.^13,14^

Optical microscopy techniques are commonly used to examine cellular stress responses. Fluorescence microscopy is particularly useful for analysing metabolism of live cells by employing functional fluorescent molecules to selectively label biomolecules. Continued advancements have been made towards the development of molecular probes and assessing their analytical applications, including those for evaluating mitochondrial metabolism^15^ and autophagic activity.^16–19^ However, fluorescence labelling may interfere with the inherent functions of biomolecules, which could affect the reliability of results.^20–22^ Furthermore, high labelling efficiency and specificity are essential for accurate fluorescence measurements, as low specificity can lead to increased background noise.^23^ Serum-free cultures are generally used for fluorescent labelling of live cells to enhance the labelling efficiency and specificity. However, because serum is crucial for maintaining live cells, the use of serum-free cultures can cause nutritional stress and potentially compromise the reliability of the results. Therefore, it is important to exercise caution when interpreting such measurement results and to combine fluorescence microscopy with complementary measurement techniques to ensure accurate and reliable results.

Photothermal (PT) microscopy is a technique that uses the PT effect to visualise trace amounts of light-absorbing molecules with high sensitivity and spatial resolution.^24–26^ The signal intensity in PT microscopy is directly proportional to the absorbance of the sample, which allows for the analysis of sample components and characteristics *via* measurements at various excitation wavelengths.^27,28^ This technique can be combined with fluorescence microscopy to obtain complementary information. In previous studies, we demonstrated the use of dual-pump PT microscopy in visualising mitochondria and lysosomes, which play crucial roles in cellular metabolism and stress responses.^29^ Cytochrome *c*, an endogenous pigment in mitochondria, absorbs light and serves as a source of PT signal.^30^ Lysosomes are cellular organelles that contain hydrolytic enzymes for intracellular digestion. Lysosomal degradation products and their residues are believed to generate PT signals. In this study, we aimed to visualise cellular responses to glucose deprivation using PT microscopy without the use of labelling molecules. We investigated as to how stress-induced changes in these organelles can be visualised using PT microscopy. We demonstrate the application of PT microscopy for obtaining biological insights into cellular responses to glucose deprivation.

## Results and discussion

### PT imaging of live HeLa cells under glucose deprivation

Two distinct culture media were used to inhibit glucose metabolism. The first medium contained glucose and was supplemented with 2-deoxy-d-glucose (2-DG), a glucose analogue that cannot be metabolised *via* the glycolytic pathway. The competition between 2-DG and glucose causes inhibition of glucose metabolism.^31^ The second medium was a glucose-free medium. Both conditions are associated with decreased glucose metabolism, but their effects on the cells and underlying mechanisms are believed to be different.^32,33^ The experiments were performed on a cervical cancer cell line, HeLa. The cells were incubated in the respective treatment medium for up to 4 days. Under glucose deprivation conditions, the growth rate of the cells decreased, and a subset of cells died and detached from the surface of culture vessel. Before microscopic observation, the medium was refreshed to remove the dead cells, and only viable cells were imaged.

**Fig. 1:**
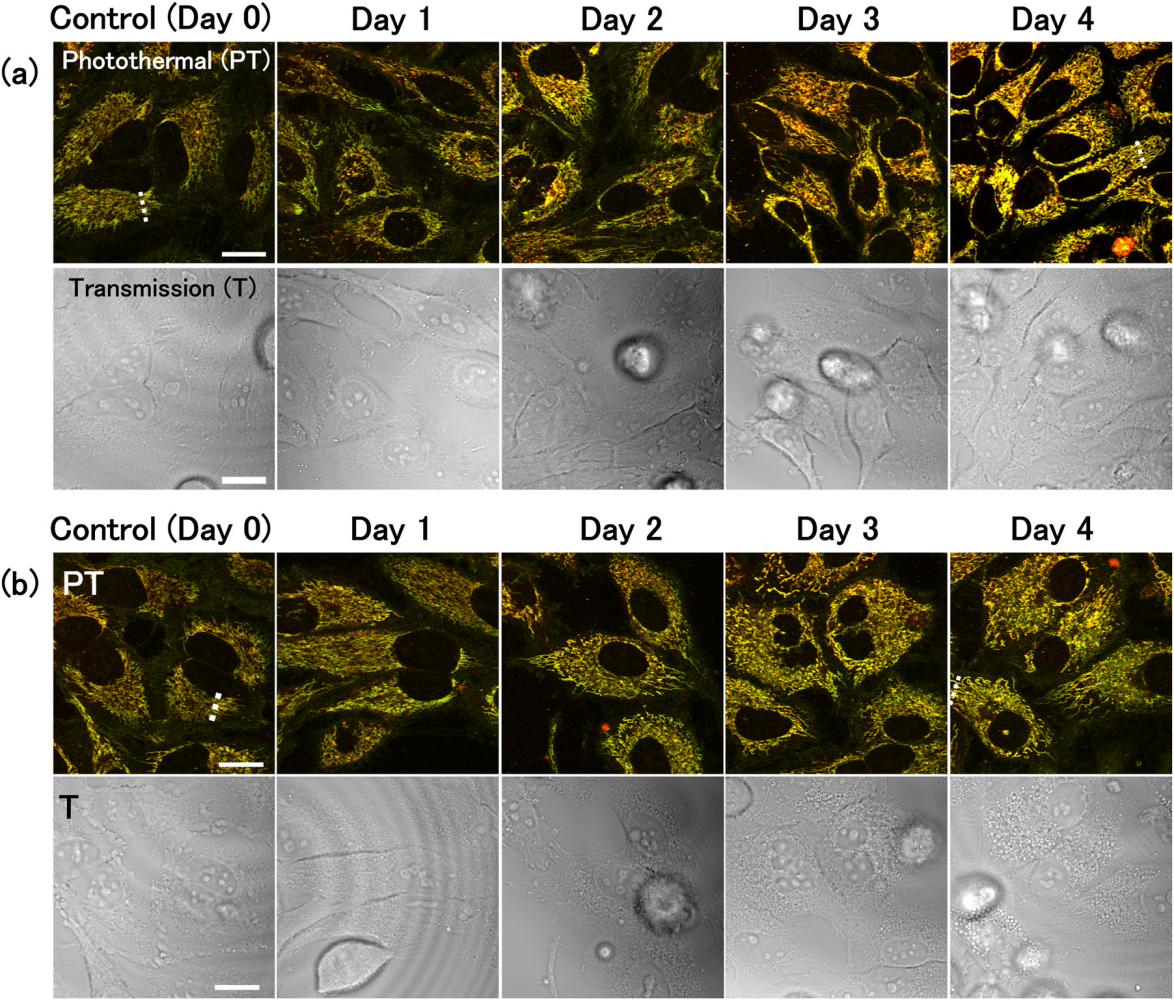
Photothermal (PT) and transmission (T) imaging of live HeLa cells under glucose deprivation conditions. Cells were cultured for 0–4 days in (a) 2-DG-added medium or (b) glucose-free medium. The images consisted of 1000 × 1000 pixels and the image acquisition time was 11 s. Scale bars, 20 μm.

PT images of HeLa cells were captured 0–4 days after replacement with either 2-DG-supplemented or glucose-free medium (Fig. 1). Two-colour images were obtained by combining two images acquired simultaneously with pump light at the wavelengths of 520 nm (green) and 640 nm (red). Transmission images were also acquired by measuring the intensity of the transmitted probe light. PT signals were absent from the nuclei, resulting in circular black shadows in the images. Mitochondria were observed as filamentous structures surrounding the nuclei. Because the signal intensity at 520 nm is slightly greater than that at 640 nm, mitochondria appeared yellowish-green in the overlay image. Some mitochondria could be discerned in the transmission images owing to their higher refractive index compared to that of cytoplasm^34^ (Fig. S1, ESI†). Lysosomes were visualised as small brown punctate structures within the mitochondrial network. Both mitochondria and lysosomes exhibited characteristic alterations under glucose deprivation conditions. In contrast, HeLa cells cultured in normal medium containing glucose (without 2-DG) for 0–4 days showed no significant changes (Fig. S2, ESI†).

### Increase in mitochondrial PT signal intensity

**Fig. 2:**
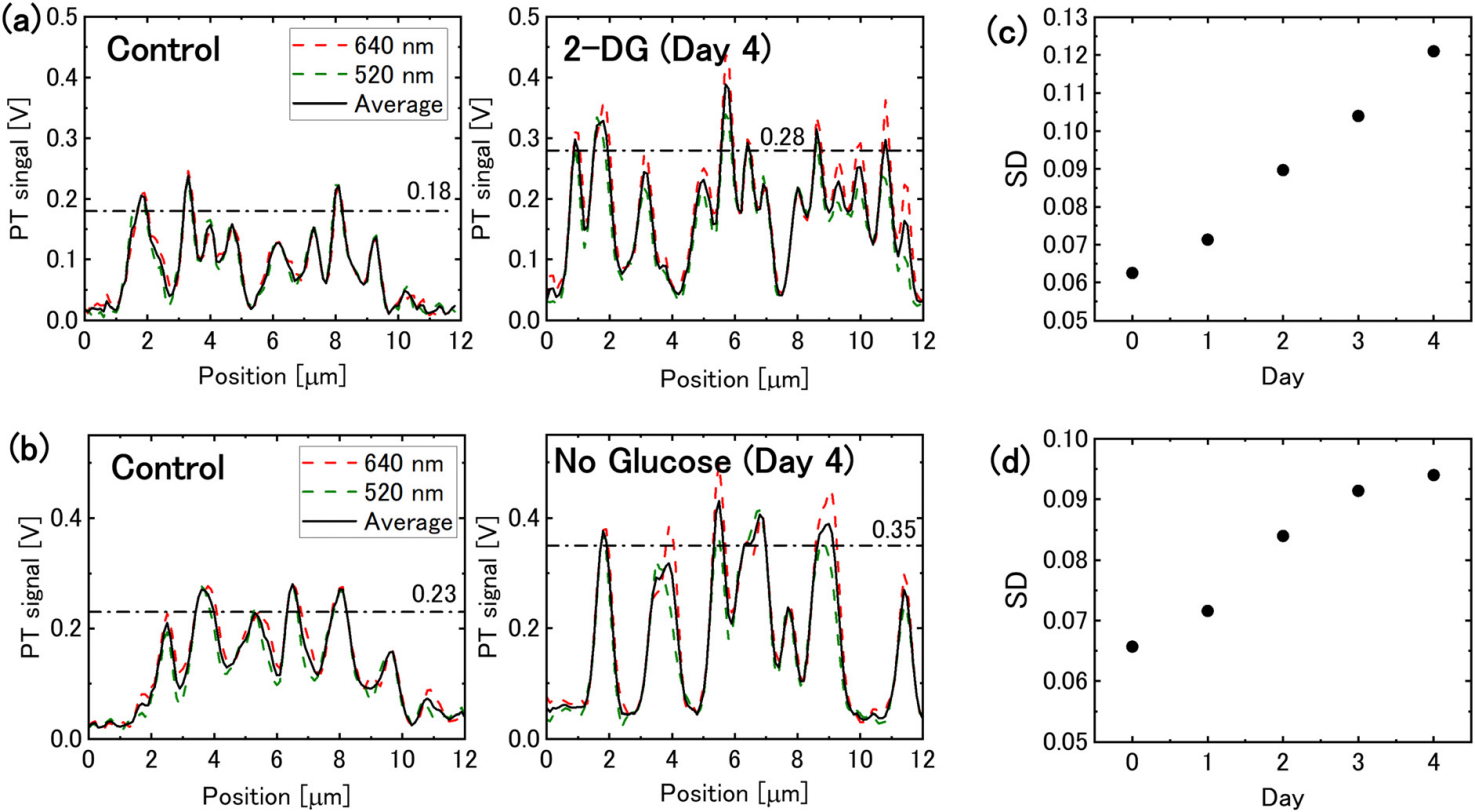
Mitochondrial photothermal (PT) signal under glucose deprivation conditions. (a) Intensity profiles of the mitochondrial PT signal for cells incubated in 2-DG-added medium for 0 and 4 days, corresponding to the dotted lines in Fig. 1(a). The dashed-dotted lines represent the average peak values. (b) Intensity profiles of the mitochondrial PT signal for cells in glucose-free medium (dotted lines in Fig. 1(b)). (c) and (d) Temporal changes in the mitochondrial PT signal evaluated on the basis of the standard deviation (SD) of the pixel values in the PT images for cells in (c) 2-DG-added medium and (d) glucose-free medium.

The PT signal intensity of mitochondria increased in both 2-DG-supplemented and glucose-free media. Fig. 2(a) and (b) illustrate the intensity profiles of mitochondrial PT signal. Following 4 days of glucose deprivation, the peak signal intensity increased by approximately 1.5-times. This increase enhanced the image contrast, allowing the mitochondrial PT signal to be quantified as the standard deviation of the image pixel values. The standard deviations of pixel values for each image indicate that the mitochondrial PT signal increased over time under glucose-deprivation conditions (Fig. 2(c) and (d)).

The density of the inner membrane cristae has been reported to increase in response to nutrient deprivation for optimising the efficiency of energy production in mitochondria.^5^ The observed increase in mitochondrial PT signal intensity is believed to have resulted from increase in crista density. An increase in the density of mitochondrial cristae potentially results in an increase in the number of electron transport complexes.^35^ Glucose deprivation leads to the upregulation of mitochondrial cytochrome *c* oxidase II, which is essential for the formation of respiratory chain complex IV.^36^ Such respiratory chain complexes, which contain heme iron, are believed to generate PT signals. Furthermore, the lipid membrane that forms mitochondrial cristae is thought to exist in a liquid crystalline state. Lipids and liquid crystals have been reported to exhibit greater temperature-dependent changes in their refractive indices (|d*n*/d*T*| = ∼6 × 10^−4^ K^−1^ for lipid droplets^37^ and ∼3 × 10^−3^ K^−1^ for nematic liquid crystals^38^) than that of water (1 × 10^−4^ K^−1^). Since the PT signal intensity is proportional to |d*n*/d*T*|, it is assumed that an increase in crista density enhances PT signal intensity *via* an increase in |d*n*/d*T*|.

Transmission electron microscopy is typically employed to examine the structure of cristae because of its high spatial resolution requirements; however, it cannot be used on living cells. A recent breakthrough has enable the observation of mitochondrial cristae in living cells using stimulated emission depletion super-resolution fluorescence microscopy, but this technique requires a specific dye capable of labelling the inner mitochondrial membrane and the ability to withstand exposure to intense laser light.^39^ Although direct observation of the cristae structure is not possible with the spatial resolution (approximately 0.3 μm) of the PT microscope, this study demonstrates that the crista density can be assessed based on the PT signal intensity. Observation of mitochondria using PT microscopy is less susceptible to photobleaching,^29^ ensuring reliable assessments. Fig. 2(a) and (b) show a slightly greater increase in signal intensity at 640 nm compared with that at 520 nm, which may be related to environmental changes affecting hemeproteins, such as the formation of respiratory supercomplex.^40^ For more detailed analysis, photoelectron correlation microscopy, which combines PT and transmission electron microscopy, is suggested.

### Relationship between lysosomal PT signal and autophagic activity

After 1 day of incubation in the 2-DG-supplemented medium, the PT signal intensity of the lysosomes increased (Fig. 1(a) and Fig. S3, ESI†). Nutrient deprivation is well-known to induce autophagy. If the substances delivered to the lysosome *via* autophagy are the source of the PT signal, the PT signal intensity of the lysosome should be related to the autophagic activity.

**Fig. 3:**
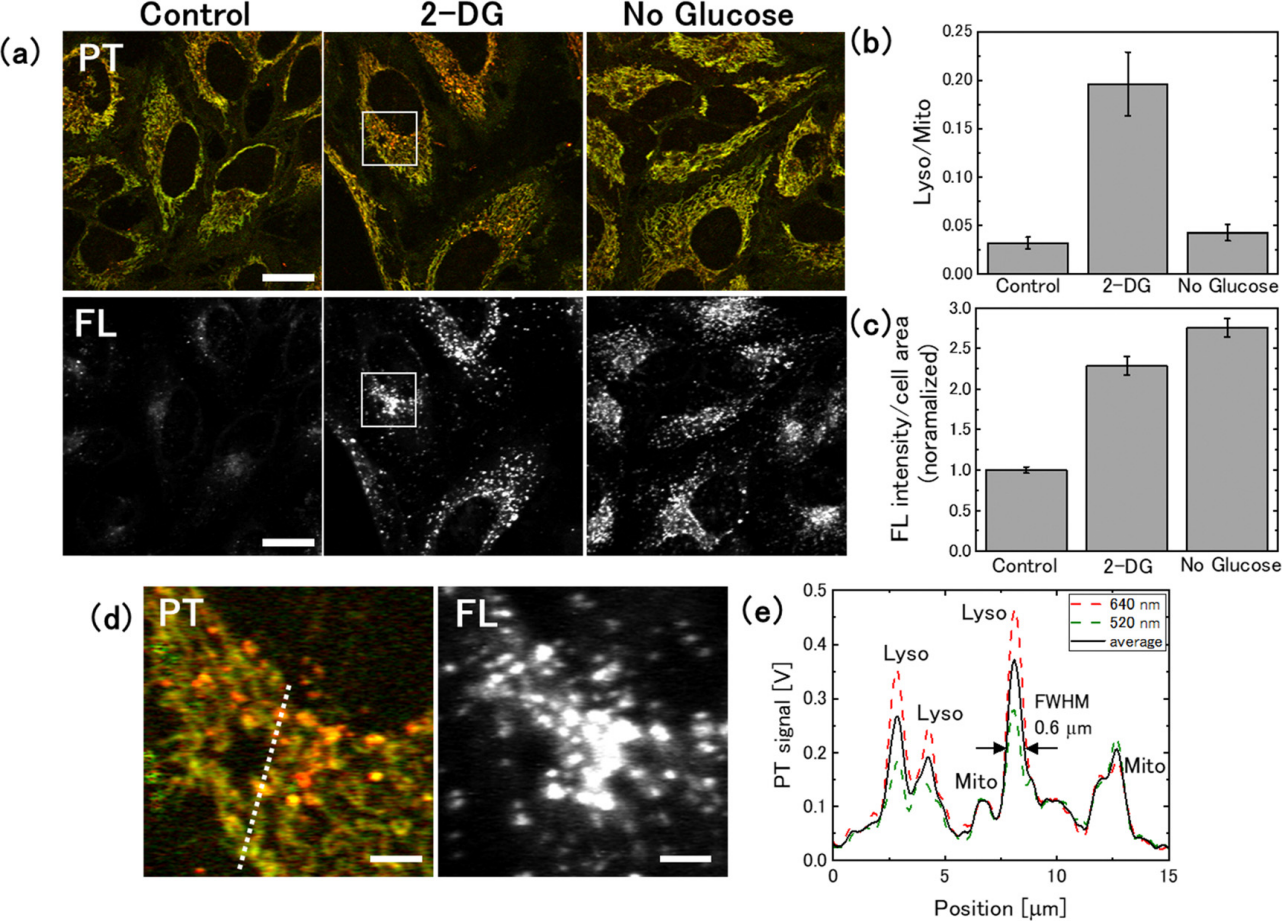
Relationship between lysosomal photothermal (PT) signal and autophagic activity under glucose deprivation conditions. (a) PT and fluorescence images of live HeLa cells stained with DAPGreen. Cells were cultured in 2-DG-added or glucose-free medium for 1 day. Scale bar, 20 μm. (b) The ratio of lysosomal-to-mitochondrial (Lyso/Mito) PT signal for glucose-deprived cells. Averages of Lyso/Mito were obtained from 48 cells for each medium; The bars represent the standard errors. (c) Evaluation of the fluorescence signal from 48 cells, with the error bars representing the standard error of the mean. (d) Magnified image of the square in (a) showing the spatial co-localisation of lysosomal PT signals with fluorescence signals. Scale bar, 4 μm. (e) Intensity profiles along the dotted line in (d).

To evaluate the relationship between the intensity of the lysosomal PT signal and autophagic activity, we stained cells with fluorescent dyes that label autophagosomes and autolysosomes,^17^ and acquired PT and fluorescence images using a PT microscope equipped with a confocal fluorescence detection system (Fig. 3(a)). In this procedure, a fluorescent image was initially obtained by irradiating the sample with excitation light at 450 nm, and subsequently switching the light sources and observing the same area using the PT microscope. To quantify the lysosomal PT signal from the images, the lysosomes and mitochondria in each cell were segmented based on their hue (the ratio of signal intensity at 520 and 640 nm), and the ratios of their integrated values (Lyso/Mito) were obtained from 48 cells for each medium (Fig. 3(b)). The Lyso/Mito ratio increased by an average of 6.3-fold in the 2-DG-supplemented medium compared with that in the control medium. The fluorescence intensity per cell area increased by 2.3-fold, indicating autophagy activation (Fig. 3(c)). Furthermore, the lysosomal PT signal co-localised with the fluorescence signal (Fig. 3(d)). The size of the lysosomes was approximately 0.6 μm in the full width at half maximum (FWHM), which was consistent with the lysosome size observed in the control medium (Fig. 3(e) and Fig. S4, ESI†). The time-lapse measurements revealed that the lysosomes in the 2-DG medium showed high motility (Movie S1, ESI†), which was also similar to that observed for the lysosomes in the control medium.^29^

In contrast, the cells cultured in glucose-free medium for 1 day showed no significant change in the lysosomal PT signal intensity (Fig. 3(b)). However, fluorescence imaging confirmed the up-regulation of autophagy (Fig. 3(c)). This absence of change in the lysosomal PT signal intensity can be attributed to the lysosomal dysfunction occurring due to glucose deprivation.

Previous studies have shown that the degree of lysosomal acidification is reduced in cells cultured in glucose-free or low-glucose medium, thereby decreasing the activity of degradative enzymes and blocking the autophagic flux.^41,42^ In this case, substances delivered to the lysosomes remain undegraded, leading to lysosomal enlargement. Indeed, after 4 days of replacement with glucose-free medium, an accumulation of hypertrophic lysosomes in the cells was observed (Fig. S5, ESI†). These hypertrophic lysosomes can be identified in the transmission images. In normal lysosomes, substances transported *via* autophagy are degraded and subsequently expelled; however, degradation residues accumulate, leading to the formation of lipofuscin pigments.^43^ The strong lysosomal PT signal observed in cells cultured in 2-DG-supplemented medium is believed to originate from degradation residues. In glucose-free medium, the absence of marked changes in the lysosomal PT signal intensity is attributed to the reduced accumulation of degradation residues, which is presumably associated with impaired lysosomal function.

These findings indicate that PT microscopy can be used to assess the activity of the autophagy-lysosomal system by detecting accumulated substances within lysosomes. It should be noted, however, that the results are not necessarily consistent with those obtained with conventional assessment techniques using fluorescent molecules to tag autophagosomes or autolysosomes.^16,17^ By combining these two measurement approaches, complementary information can be obtained and a comprehensive evaluation of the autophagy-lysosome system can be conducted.

### Multi-wavelength PT analysis of substances within lysosomes

**Fig. 4:**
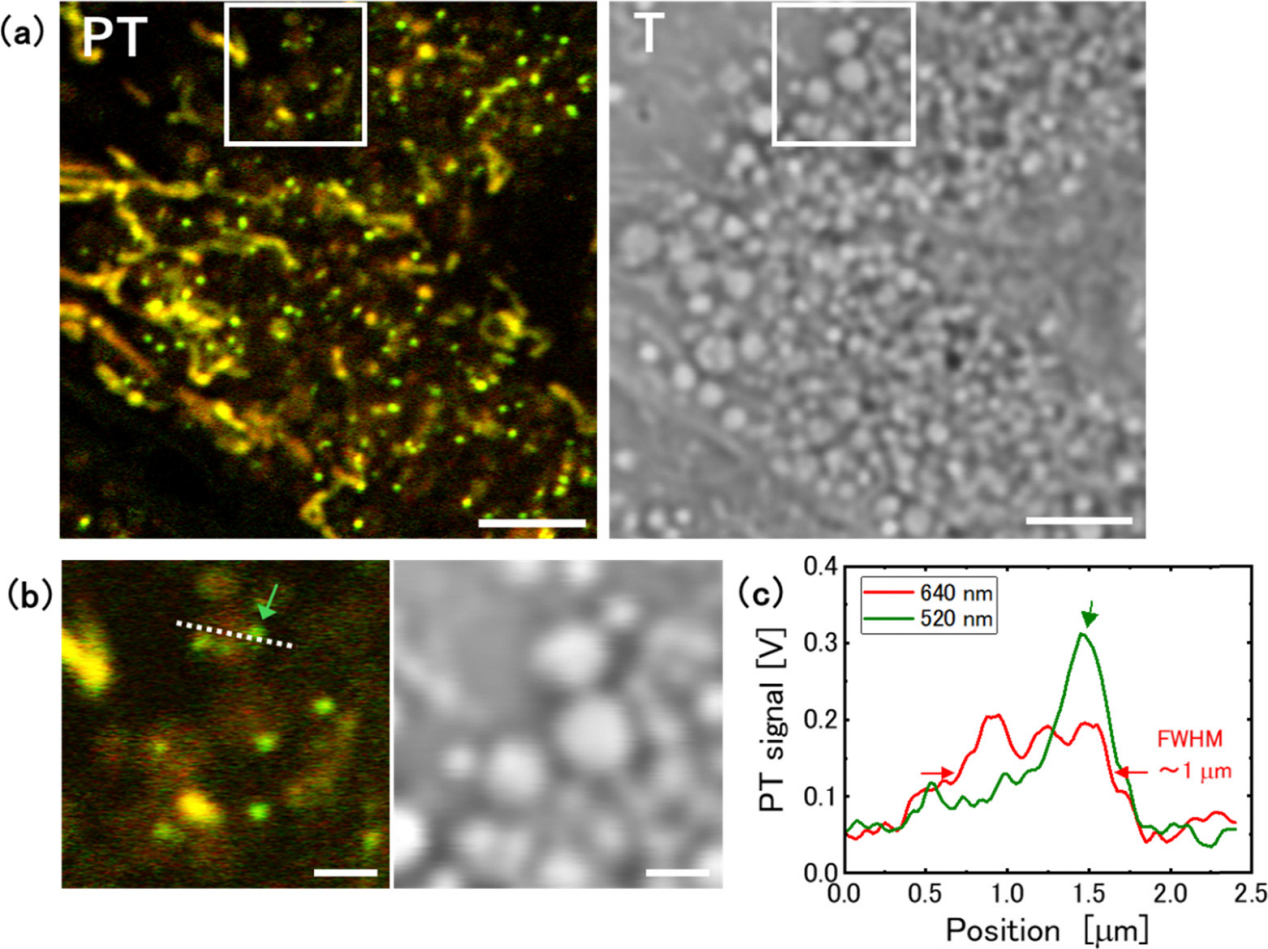
Hypertrophic Lysosomes in a cell cultured in glucose-free medium for 4 days. (a) Photothermal (PT) and transmission (T) images of the same area. Scale bar, 5 μm. (b) Magnified image in the squares in (a). Scale bar, 1 μm. (c) Intensity profiles along the dotted line in (b).

PT microscopy enables the examination of substances within lysosomes. Fig. 4(a) and (b) show the PT and transmission images of hypertrophic lysosomes in a cell cultured in glucose-free medium for 4 days. The lysosomes in glucose-free medium enlarged to their maximum size of approximately 1 μm (Fig. 4(c)). Notably, green punctate structures were observed within the lysosomes. The punctate structure showed a signal intensity at 520 nm that was twice as high as the signal intensity at 640 nm. Such punctate structures were absent in cells cultured in both the control and 2-DG-supplemented medium. In addition, weak brown PT signals were detected within the lysosomes. The molecular composition and source of these substances is discussed in the following section.

#### The green punctate structures in lysosomes

**Fig. 5:**
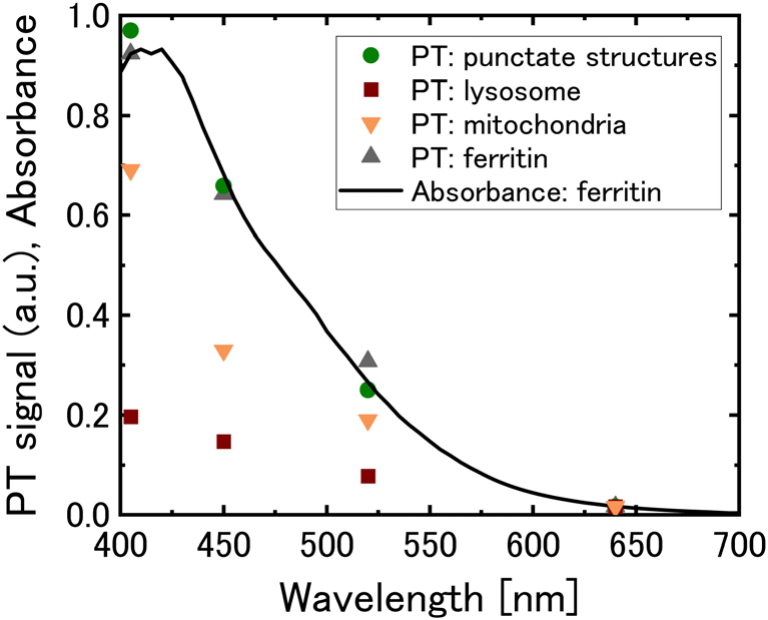
Wavelength dependence of the photothermal (PT) signals obtained for the green punctate structures (circles), brown signal in lysosomes (squares), mitochondria (inverted triangles), and aqueous solution of ferritin (triangles). The solid line represents the absorption spectrum of aqueous solution of ferritin. The PT signals are normalised with respect to the absorbance of ferritin at 640 nm.

To identify the molecular species in the green punctate structures, we used a four-wavelength PT microscope with pump light at 405, 450, 520, and 640 nm and examined the dependence of the signal intensity of the intracellular organelles on the excitation wavelength (Fig. 5). The punctate structures showed stronger signal intensity at shorter wavelengths, and their wavelength-dependence was consistent with that of ferritin. Ferritin, a protein that forms a spherical cage-like structure, stores ferric ions safely within its complex. When cells require iron, they break down ferritin through autophagy and convert the stored ferric ions into their divalent form for use.^44^ In a recent study, ferritin was shown to undergo selective degradation *via* autophagy by forming condensates through liquid–liquid phase separation.^45^ The observed punctate structures are believed to be ferritin clusters that have been delivered to lysosomes. The time-lapse measurement shows that these ferritin clusters adhere to the lysosomal surface (Movie S2, ESI†).

Iron is an essential element for various metabolic and physiological functions of living organisms. However, excess iron can lead to the production of harmful free radicals that damage biological molecules. Ferroptosis, *i.e.*, the iron-mediated cell death, has attracted attention as a new target for cancer therapy.^46^ In a recent study, an iron-sensitive fluorescent probe was used to demonstrate the accumulation of iron in lysosomes under glucose deprivation conditions, followed by cell death *via* ferroptosis.^42^ Our findings indicate that iron is stored in lysosomes in the form of ferritin clusters. As mitochondria are the main organelles that use intracellular iron, the accumulation of ferritin may also be related to metabolic changes in mitochondria. In this context, PT microscopy could provide a new perspective in iron metabolism research.

#### Brown lysosomal PT signals

ER is the organelle responsible for the folding of proteins to form the correct three-dimensional structure. Glucose deprivation causes abnormal protein folding in ER, resulting in the accumulation of denatured proteins. Denatured proteins tend to aggregate and are highly toxic to cells. In addition to the ubiquitin–proteasome system, the autophagy-lysosome system is activated to remove and degrade such denatured proteins.

**Fig. 6:**
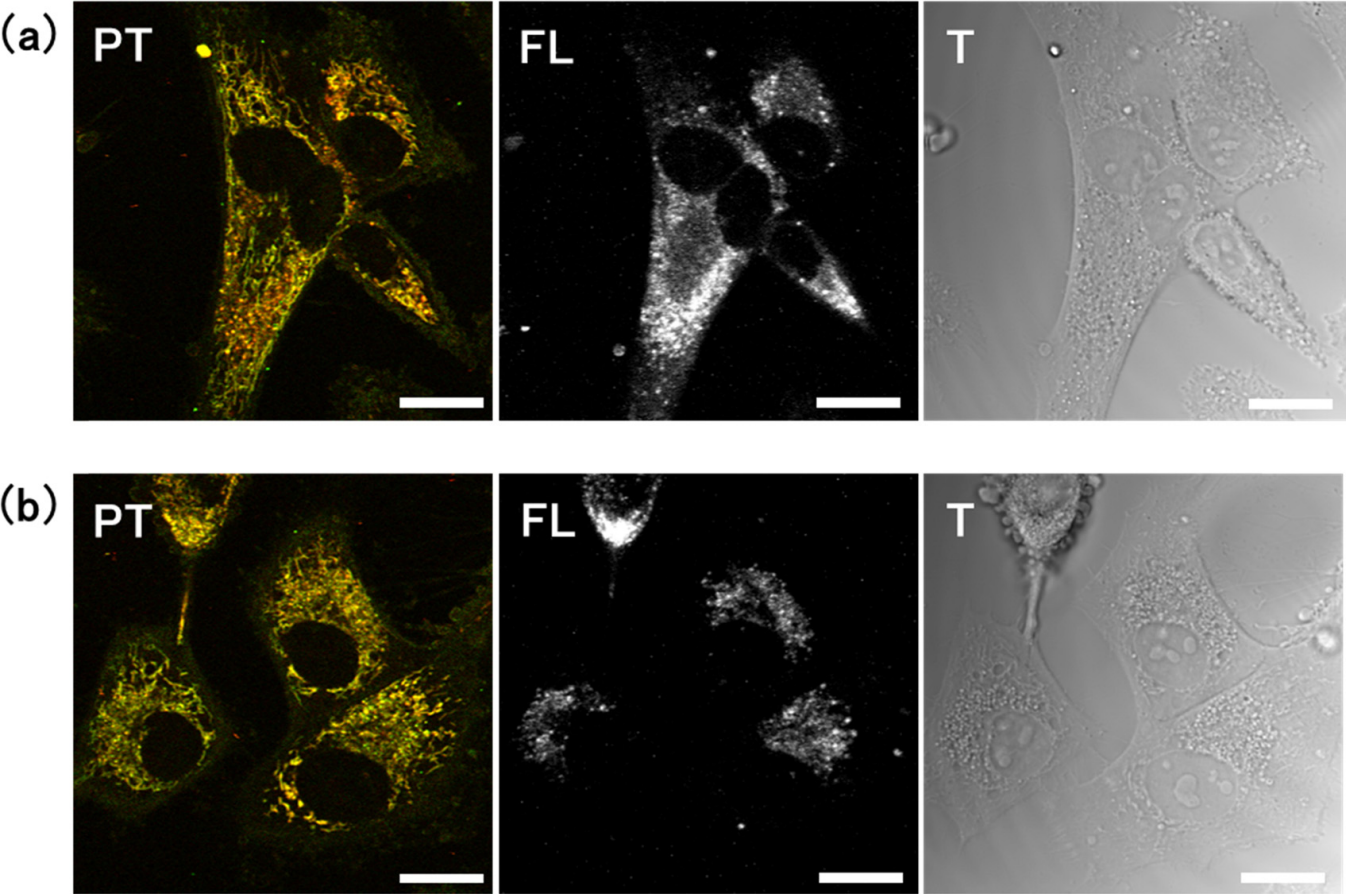
Identification of endoplasmic reticulum (ER)-derived protein signals using the photothermal (PT) technique. Photothermal (PT), fluorescent (FL), and transmission (T) images were obtained for cells labelled with a fluorescent marker for ER-associated proteins. The cells were cultured in (a) 2-DG-supplemented or (b) glucose-free medium for 4 days. Scale bar, 20 μm.

Cells cultured in glucose-free medium for 4 days showed brown signals from lysosomes. Similarly, cells cultured in 2DG-supplemented medium for 4 days showed brown PT signals within the lysosomes and in their surrounding regions. To verify that these brown PT signals were generated by ER-derived proteins, we acquired PT and fluorescence images of cells with ER-associated proteins specifically tagged using fluorescent dyes (Fig. 6). The ER-labelled cells were cultured for 4 days in both glucose-free and 2-DG-supplemented media. The brown PT signal in 2-DG-cultured cells overlapped with the fluorescence signal (Fig. 6(a)). Similarly, in the cells cultured in the glucose-free medium, the brown (or yellowish-brown) PT signal coincided with the fluorescence signal (Fig. 6(b)). In this case, the hypertrophic lysosomes visible in the transmission image co-localised with the fluorescence signal. These observations indicate that the brown lysosomal PT signals originate from proteins that had been transported from the ER for degradation.

While normal proteins do not absorb visible light, those bound to transition metal ions do, making them detectable using PT microscopy. Transition metal ions, such as ions of iron and copper, contained in cells have affinity for proteins and are involved in their denaturation and aggregation. In neurodegenerative diseases, such as Alzheimer's disease, abnormal protein aggregates accumulate in neurons; these aggregates contain a large amount of transition metal ions.^47^ To confirm whether protein aggregates containing transition metal ions can actually be a source for PT signals, we added iron and copper ions to albumin solution in test tubes to induce aggregation, and observed the samples with the four-wavelength PT microscope (Fig. S6, ESI†). The results showed that the wavelength dependence of the PT signal intensity from the aggregates was dependent on the metal ion species, confirming that the metal ions in the aggregates were responsible for the PT signal as light absorbers.

Measurements of lysosomes using the four-wavelength PT microscope showed that the brown PT signal of lysosomes exhibited a larger signal at shorter wavelengths, comparable to those of iron ion-containing proteins, including ferritin, mitochondria, and the iron ion-aggregated albumin (Fig. 5 and Fig. S6c, ESI†). However, the signal showed a relatively flat wavelength dependence, suggesting that lysosomes contain molecules other than iron ions, such as other transition metal ions or lipid peroxides, as optical absorbers. More detailed analysis is needed to elucidate the molecular species contained in lysosomes.

### PT imaging of live HeLa cells under serum starvation conditions

The culture medium is typically supplemented with serum, which contains a variety of components that are essential for cell growth. Serum starvation is one of the most commonly used techniques to study the molecular mechanisms of cellular stress response.^48^ Serum starvation is also used as an experimental model to replicate pathological conditions, such as poorly vascularised tumors,^49,50^ myocardial infarction,^51^ and stroke.^52^

**Fig. 7:**
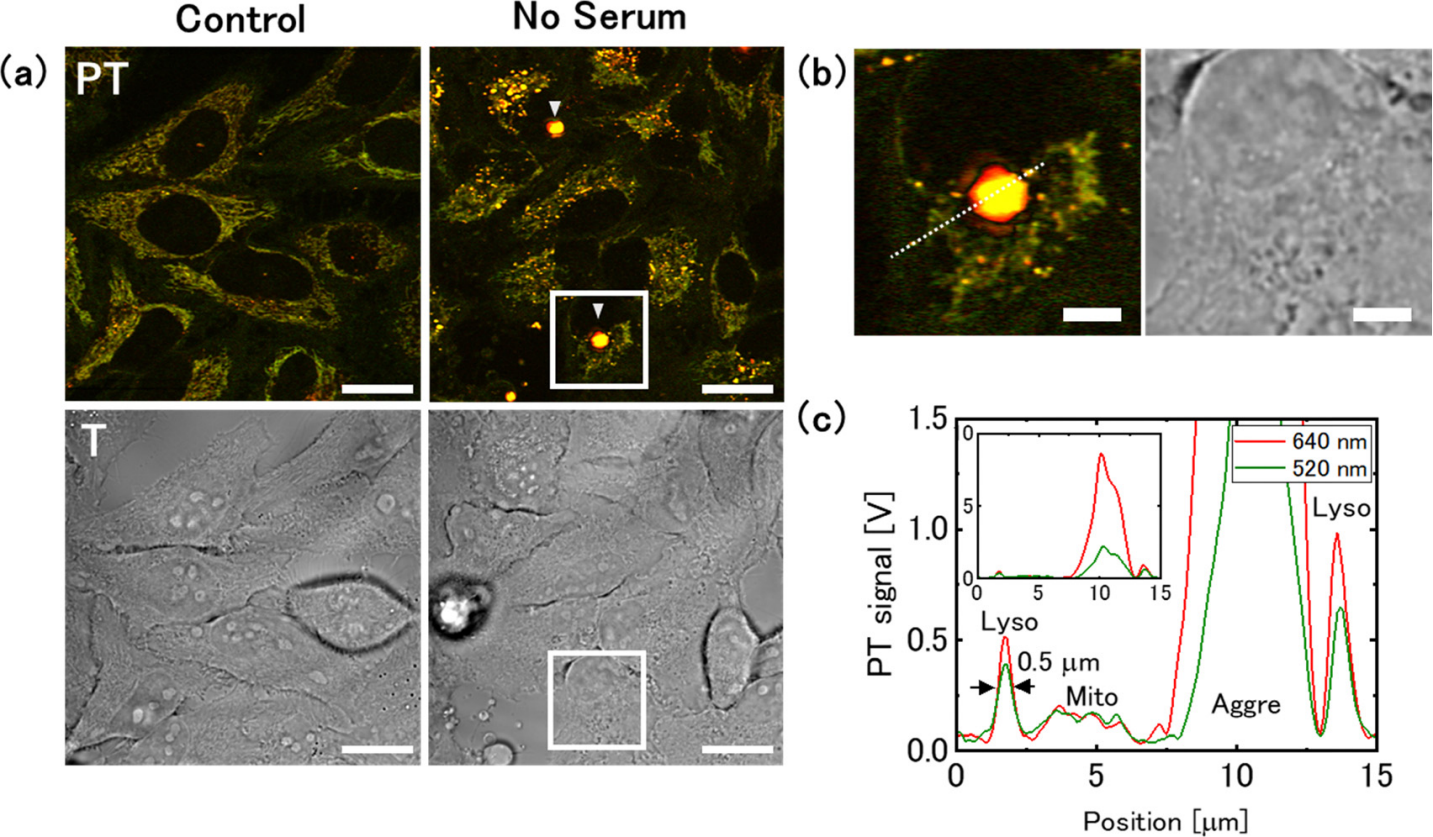
Increase in the intensity of lysosomal photothermal (PT) signals and formation of aggresome-like structures under serum starvation conditions. (a) PT and transmission (T) images were obtained from the same region. The arrow-heads indicate aggresome-like structures. Scale bars, 20 μm. (b) Magnified images of the squares in (a). Scale bar, 5 μm. (c) Intensity profiles from the lysosome, mitochondria, and the aggresome-like structure, corresponding to the dotted-line in (b).

**Fig. 8:**
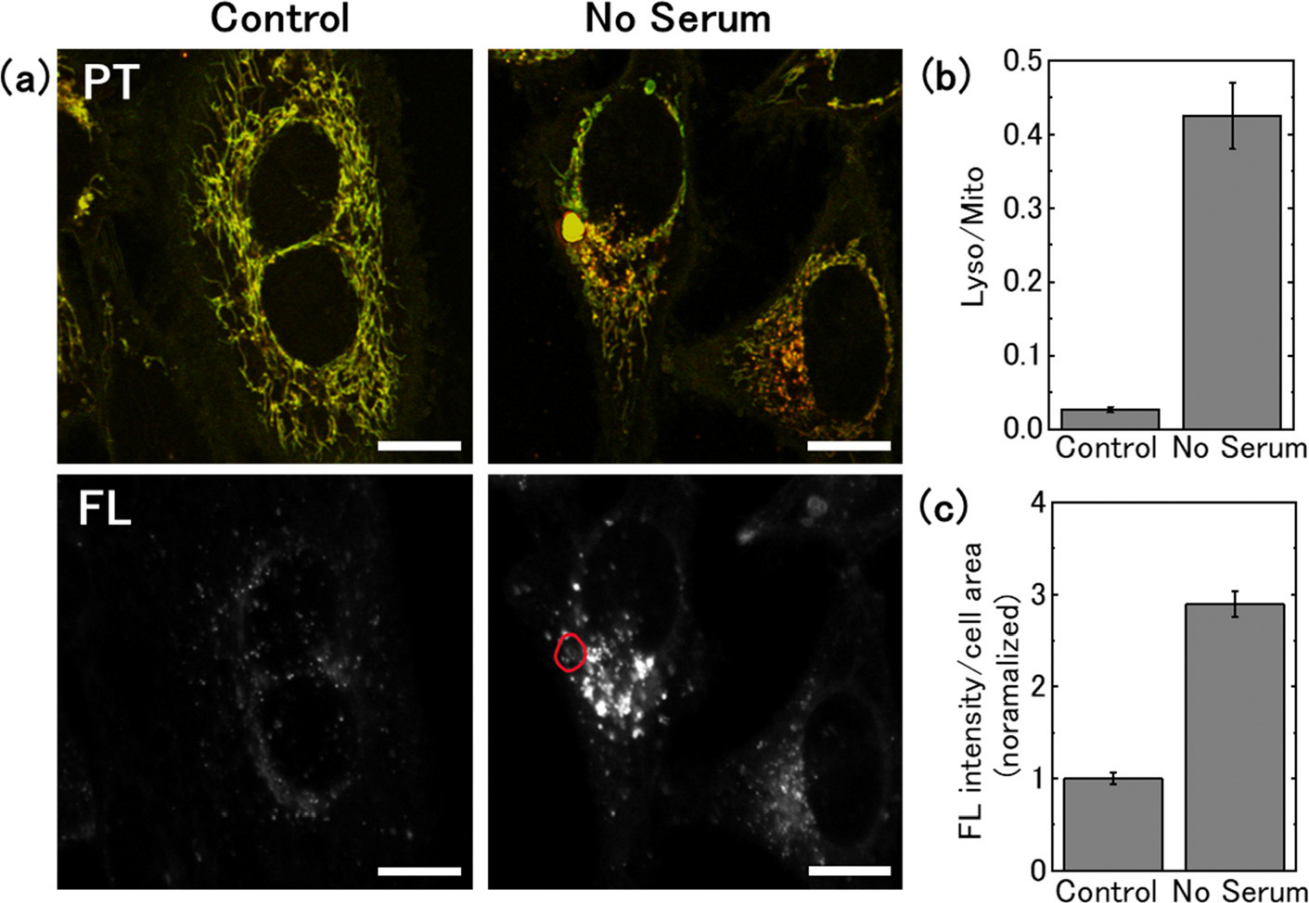
Relationship between lysosomal photothermal (PT) signal and autophagic activity under serum starvation conditions. (a) PT and fluorescent (FL) images of cells stained with DAPGreen. Cells were cultured in serum-free medium for 1 day. The area surrounded by the red line in the FL image indicates the location of the aggregate-like structure observed in the PT image. Scale bars, 20 μm. (b) Averages of the Lyso/Mito ratio from 58 (control) and 36 (no serum) cells, with the bars representing the standard errors. (c) Evaluation of the fluorescence signal from the cells, with the bars representing standard error of the mean.

We performed PT imaging of live cells cultured in serum-free medium and compared the results with those obtained in glucose deprivation (Fig. 7). As many cells in serum-free medium die a few days after medium replacement, we selected cells after 1 day of medium replacement for the measurement. In the serum-free medium, no significant change in the mitochondrial signal was noted; however, a significant increase in the lysosomal PT signal was observed (Fig. 7(a) and (b)). The lysosomal size was 0.5 μm in FWHM, consistent with that observed in the control medium (Fig. 7(c)). Fluorescence and PT imaging of cells stained with an autophagy detection dye showed that serum deprivation induced autophagy, with the fluorescent signal overlapping the lysosomal PT signal (Fig. 8). This result is consistent with that obtained from cells cultured in 2-DG supplemented medium (1 day), suggesting that PT microscopy can be used to assess the activity of the autophagy-lysosome system regardless of the specific nutrient deficiency. The intensity of the lysosomal signal (Lyso/Mito) in the serum-free medium was approximately twice that in the 2-DG-supplemented medium. This difference may be due to increased transport of substrates to lysosomes and increased accumulation of degradation residues under serum starvation conditions.

Furthermore, a structure producing a strong PT signal was seen around the nuclei (Fig. 7(a) and 8). The PT signal from this structure was approximately 10-times larger than that of the lysosome (Fig. 7(c)). This may be attributed to an aggresome in which the aggregated protein was sequestered. Aggresomes are typically formed near the nucleus when the accumulation of protein aggregates exceeds the proteolytic capacity of the cell. The wavelength dependence of the signal intensity from the aggresome-like structure is larger at shorter wavelengths, but is relatively flat, as is the case with lysosomes (Fig. S7, ESI†). Because aggresome-forming substances are transported to lysosomes *via* autophagy for degradation, they are presumed to share common components with substances that accumulate in the lysosomes.

**Fig. 9:**
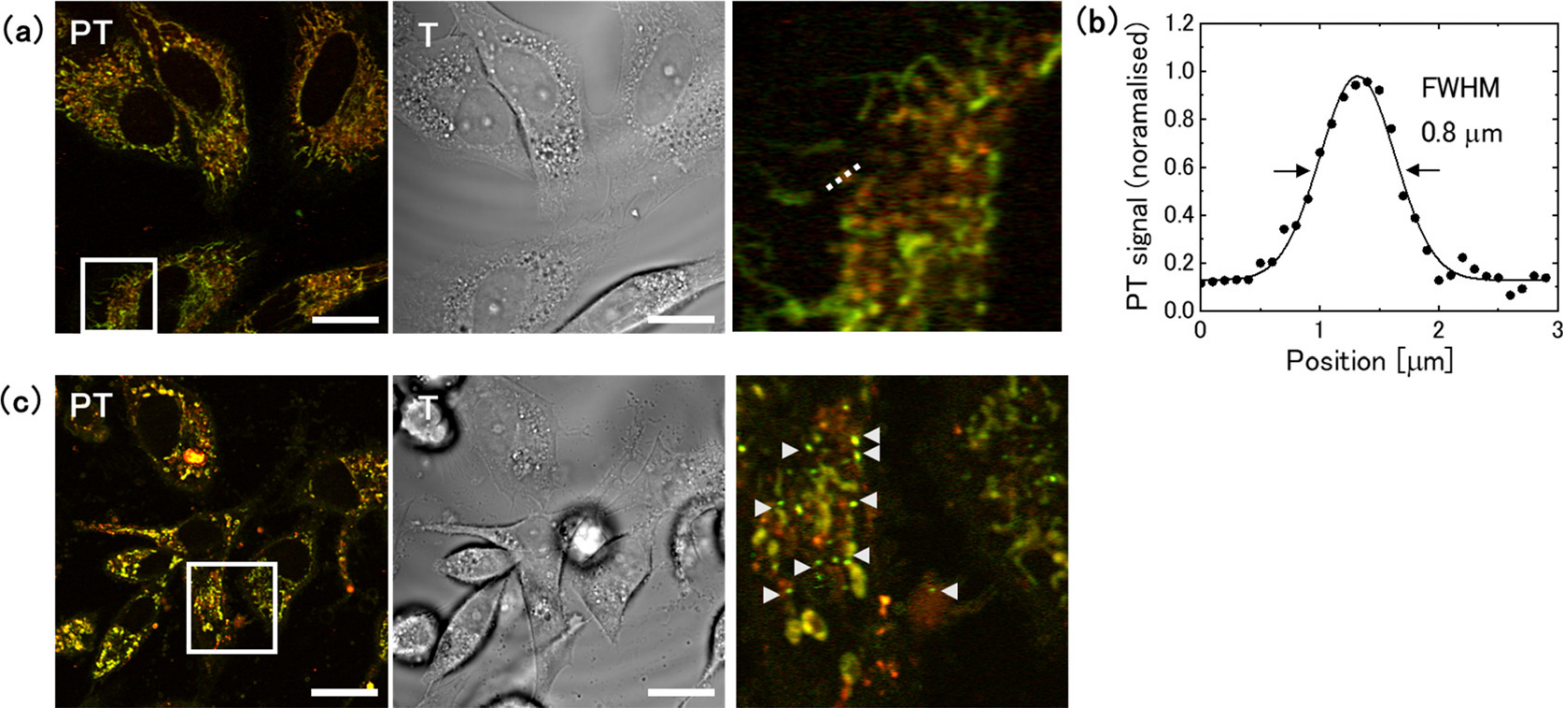
Photothermal (PT) imaging of the effect of chloroquine on cells. (a) PT and transmission (T) images of cells cultured in media containing chloroquine with serum. Scale bars, 20 μm. (b) Intensity profile along the dotted line in (a) showing a lysosomal size of 0.8 μm in the full width at half maximum (FWHM). The solid line shows the fitting curve using a Gaussian function. (c) PT and T images of chloroquine-treated cells cultured in serum-free medium. The arrow-heads indicate the ferritin clusters.

To investigate how decreased lysosomal degradation capacity affects cellular stress response, the cells were treated with chloroquine (Fig. 9). Chloroquine elevates lysosomal pH and inhibits the activity of degrading enzymes,^53^ thereby simulating physiological conditions prevalent in glucose-deprived cells. Initially, cells in the serum-containing medium were observed after chloroquine addition (Fig. 9(a)). After 24 h of chloroquine treatment, cells exhibited an accumulation of brown lysosomes. The lysosomal size was 0.8 μm in FWHM, approximately 1.6-times larger than that in the control medium (Fig. 9(b)). These observations are consistent with the lysosomal characteristics observed in cells cultured in glucose-free medium for 4 days, although ferritin was absent. The time-lapse measurements revealed the reduced mobility of these hypertrophic lysosomes (Movie S3, ESI†). Next, we observed the cells cultured in serum-free medium supplemented with chloroquine (Fig. 9(c)). The intense nutrient stress resulted in cell shrinkage and mitochondrial fragmentation. The intensity of the PT signal in the lysosomes was less pronounced in comparison to that under serum starvation without chloroquine. Green punctate structures, which are believed to be ferritin, were observed. These results indicate that nutritional deficiency and lysosomal impairment are associated with the accumulation of ferritin clusters.

### Technical challenges in measuring cellular stress responses using PT microscopy

PT microscopy typically uses lasers of different wavelengths for the pump and probe beams, which leads to misalignment of the focal positions due to chromatic aberration at the edge of the image and a reduction in the signal intensity. As a result, the field of view is limited to approximately 100 μm, allowing only about five cells to be measured per image. Acquiring data from a large number of cells, such as when assessing the lysosomal signal intensity (Lyso/Mito), requires acquisition of multiple images at various sample positions, resulting in longer measurement times. In addition, this system lacks temperature control for the sample during observation, which prevents long-term monitoring of temporal changes in individual cells. Future improvements to the measurement system will be essential to address these limitations.

## Conclusions

Live imaging of HeLa cells using PT microscopy revealed that glucose deprivation led to an increase in mitochondrial PT signals, indicating that the PT signal intensity can be used to assess the density of mitochondrial cristae. Additionally, PT microscopy could be used to evaluate the activity of the autophagy-lysosome system by detecting the substances accumulated in lysosomes. Comparison of cells cultured in 2-DG-supplemented and glucose-free medium showed that the PT signal intensity depends on lysosomal degradative activity. The combination of PT, transmission (phase), and fluorescence imaging techniques allowed for the collection of complementary data, enabling a thorough assessment of the autophagy-lysosome system.

Further, using PT microscopy, we detected ferritin clusters transported to the lysosomes under glucose deprivation conditions. The ferritin clusters were also observed in cells cultured in serum-free medium supplemented with chloroquine, suggesting that nutrient deprivation and lysosomal impairment contribute to ferritin accumulation. Considering the crucial roles of ferritin, lysosomes, and mitochondria in iron regulation within cells, PT microscopy shows potential as a valuable tool for studying the adaptation of the iron metabolism in cancer cells under nutrient stress conditions.

## Material and methods

### Sample preparation

HeLa cells were obtained from RIKEN BRC (RCB0539; Riken BRC Cell Bank, Japan). The cells were cultured in Dulbecco's modified Eagle medium (DMEM), with a glucose concentration of 25 mM (Nacalai Tesque, Japan), supplemented with streptomycin (100 μg mL^−1^), penicillin (100 μg mL^−1^), l-glutamine (2 mM), and 10% foetal bovine serum (FBS), which served as the control medium. The cells were incubated in a 5% CO_2_ environment at 37 °C.

For live-cell imaging, the cells were seeded in 50 mm glass-bottom dishes (MATSUNAMI, Japan). After 24 h of incubation in the glass-bottom dish, 6 mM 2-DG was added to the control medium to inhibit glucose metabolism. Similarly, after 24 h of seeding in the glass-bottom dish, the control medium was replaced with DMEM without glucose (Nacalai Tesque) to induce glucose deprivation. The composition and concentration of the glucose-free medium was the same as that of the control medium, except that it lacked glucose. After the addition of 2-DG or replacement with glucose-free medium, the cells were cultured again in 5% CO_2_ incubator at 37 °C for 0–4 days. Because phenol red, a normal component of the culture medium, absorbs visible light and causes a background in PT imaging, the medium was replaced with phenol red-free DMEM before measurement. PT imaging was performed at room temperature (22 °C) in different sessions, typically within 1 h. Different samples were prepared and used for imaging in each session.

To evaluate the autophagy activity under glucose deprivation conditions using PT and fluorescence imaging, cells seeded in glass-bottom dishes were washed with phosphate-buffered saline (PBS) and incubated in Hanks’ balanced salt solution (HBSS) containing 0.1 μM DAPGreen (Dojindo, Japan) for 30 min. The cells were then washed with PBS and incubated in 2-DG-supplemented or glucose-free medium for 24 h.

To detect ER-associated proteins in cells under glucose deprivation conditions using a fluorescence microscope, the cells in the glass-bottom dishes were washed with PBS and then stained with 0.2 μM of a fluorescent probe (ERseeing, Dojindo) in serum-free DMEM for 1 h. The composition and concentration of the serum-free medium was the same as that in the control medium, except that it did not contain serum. The cells were then washed again and cultured for 4 days in 2-DG-supplemented or glucose-free medium.

To induce serum starvation, cells in glass-bottom dishes were cultured in the serum-free medium for 24 h. To evaluate the autophagy activity under serum starvation conditions using PT and fluorescence imaging, cells were cultured in HBSS with 0.1 mM DAPGreen for 30 min. The cells were then cultured in serum-free medium for 24 h. To inhibit lysosomal degradation, Chloroquine (50 μM) was added to the control and serum-free medium, and PT images were obtained after 24 h of incubation.

Ferritin derived from horse spleen was purchased from Sigma-Aldrich. To measure the multi-wavelength PT signals and absorption spectrum of ferritin, ferritin powder was dissolved in distilled water to a concentration of approximately 10 mM. For multi-wavelength PT analysis, 10 mL of this ferritin solution was dropped onto a glass slide and sealed with a coverslip.

### PT and fluorescence imaging

The PT images were obtained using a home-built multi-wavelength PT microscope (Fig. S8, ESI†).^28^ A 785 nm single-frequency laser diode (LD) (LP785-SAV50; Thorlabs, NJ, USA) was used for probing. The two LDs at 520 and 640 nm were primarily used for pumping to avoid photodamage to the samples. The LDs at 405 and 450 nm were used in addition to 520 and 640 nm to measure the excitation wavelength dependence of cellular organelles for multi-wavelength PT analysis. For the simultaneous lock-in detection, the intensity of each pump beam was directly modulated at different frequencies. The pump beams were combined using dichroic mirrors and collimated using a single-mode fibre (P1-405BPM-FC; Thorlabs) and an off-axis parabolic mirror. The pump and probe beams were combined using a dichroic mirror (FLD755 DLP; Iridian, Canada) and directed onto the sample *via* a galvano scanner (VM500PLUS; Cambridge Technology). An oil-immersive objective lens (×40, NA 1.25; Olympus, Japan) was used to focus the beams onto a sample, yielding a spatial resolution of 0.3 μm in the lateral plane and 0.9 μm in the axial direction. The PT images were obtained by raster scanning of the collinear pump and probe beams over the sample. The transmitted beams were collected using a collection lens and directed to the home-build photodetector. A long pass filter (FELH0750; Thorlabs) was used to block the pump light. A spatially divided balanced detection scheme was implemented using a custom-made fibre bundle (Sumita Optical Glass, Japan) to improve the signal-to-noise ratio. The PT signals for each pump beam were demodulated simultaneously by frequency division multiplexing using multiple lock-in amplifiers (LI5660; NF, Japan). The time constants of the lock-in amplifiers and pixel dwell time were set to 10 μs. The amplitude signals from the lock-in amplifiers were recorded using a simultaneous-sampling data acquisition device (PCI-6110; NI, TX, USA). Transmission images were acquired simultaneously with PT images by recording the transmitted intensity of the probe beam. For live cell imaging, the pump powers incident on the sample were set to 3.9 and 15 mW at 520 and 640 nm, respectively. The probe-beam power was set to 11 mW. The images consist of 1000 × 1000 pixels and the acquisition time was 11 s. Image processing was performed using the NIH ImageJ software.^54^

Confocal fluorescence detection optics was incorporated into the PT microscope system, in which a photomultiplier tube (H5784-04; Hamamatsu) detected the fluorescence signal from the sample through a long-pass filter (FELH0550; Thorlabs) and a pinhole. The LD at 450 nm was used to excite DAPGreen and ERseeing.

### Multi-wavelength PT analysis

To investigate the wavelength dependence of the PT signals from the cellular organelles and ferritin solution, four-wavelength LDs were used for pumping with incident powers set to 1.4 mW at 405 nm and 2.8 mW at 450, 520, and 640 nm. The probe beam power was set to 11 mW. The wavelength dependence of the PT signals for cellular organelles was obtained by averaging the PT signals over their segmented regions after background subtraction. Because the PT signal intensity of the ferritin solution was nearly uniform throughout the image, the wavelength dependence of the ferritin signal was calculated from the average of all pixel values. The signal for each wavelength was normalised to the incident pump powers and multiplied by a factor obtained from single-walled carbon nanotube measurements to correct for wavelength sensitivity.^28^ Au and Ag nanoparticles were measured as test samples to confirm that the PT signals after calibration were consistent with their absorption spectra.

## Author contributions

Conceptualization: J. M.; methodology: J. M.; software: J. M.; investigation: J. M. and R. W.; visualization J. M. and R. W.; formal analysis J. M. and R. W.; writing – original draft preparation: J. M.; writing – review & editing: J. M. and K. O. All authors have read and agreed to the final version of the manuscript.

## Data availability

Data for this manuscript are available within the text or in the ESI.†

## Conflicts of interest

There are no conflicts to declare.

## Supplementary Material

CB-006-D4CB00269E-s001

CB-006-D4CB00269E-s002

CB-006-D4CB00269E-s003

CB-006-D4CB00269E-s004
