# Supplementary material for: Photothermal imaging of cellular responses to glucose deprivation
Source: RSC Chem Biol. 2025 Jan 31;6(4):571–82. doi: 10.1039/d4cb00269e (PMC11801213; doi:10.1039/d4cb00269e)
Supplement: CB-006-D4CB00269E-s004 [file CB-006-D4CB00269E-s004.pdf]

Supplementary Information (SI) for RSC Chemical Biology.
This journal is © The Royal Society of Chemistry 2025

Supporting Information for

Photothermal imaging of cellular responses to glucose deprivation

Jun Miyazaki,[*a] Ryotaro Wagatsuma [a] and Koji Okamoto [b]

[a]*Faculty of Systems Engineering, Wakayama University, Wakayama 640-8510, Japan*
[b]*Graduate School of Frontier Biosciences, Osaka University, Osaka 565-0871, Japan*

*To whom correspondence may be addressed. E-mail: jmiya@wakayama-u.ac.jp

This file includes: Figs. S1–S8

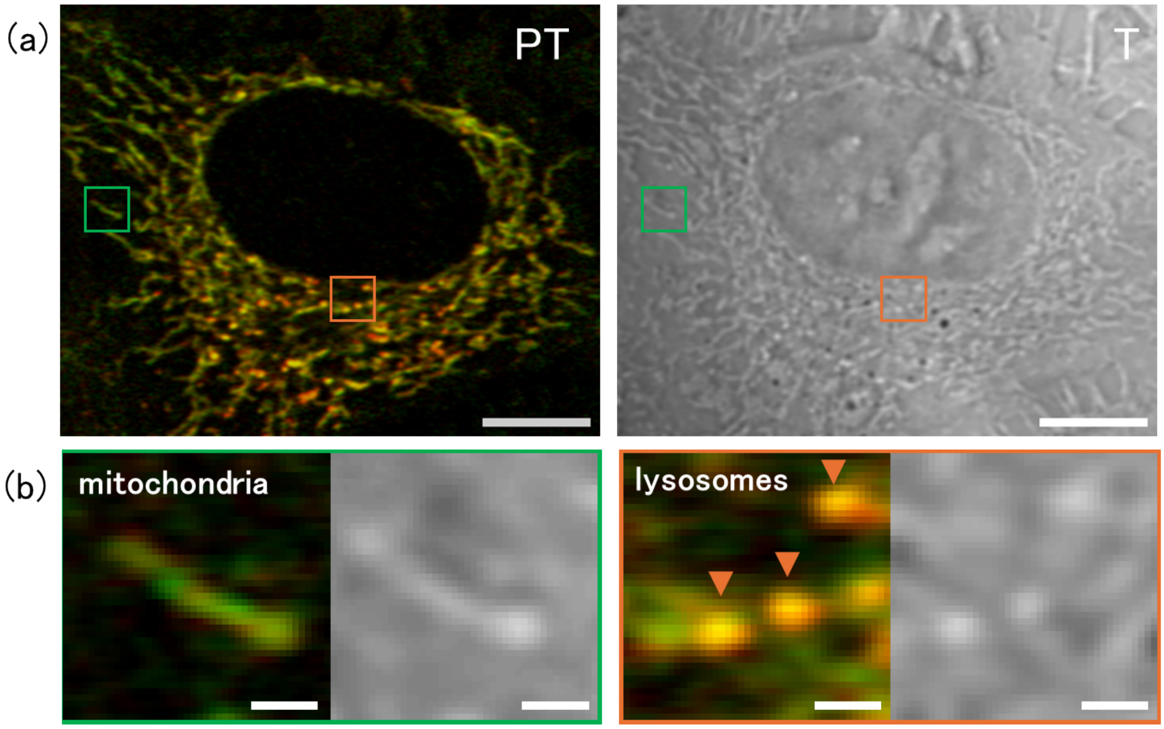

Fig. S1. (a) Photothermal (PT) and transmission (T) images of a live HeLa cell cultured in the control medium. Scale bars, 10 μm. (b) Magnified image of the squares in (a) showing mitochondria and lysosomes (arrow-heads). Scale bars, 1 μm. In the PT image, mitochondria and lysosomes appear as yellowish-green filamentous structures and small brown punctate structures, respectively. Some mitochondria can also be discerned in the transmission images owing to their higher refractive index.

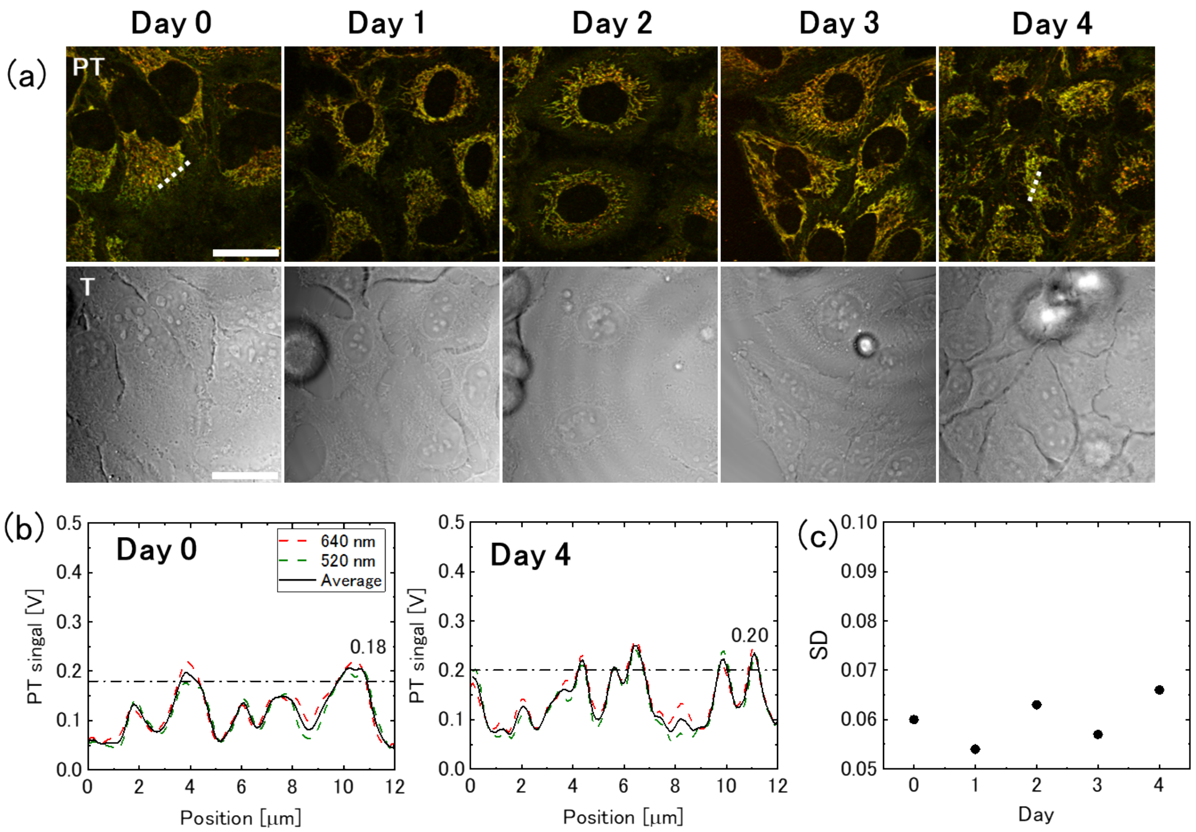

Fig. S2. (a) Photothermal (PT) and transmission (T) imaging of live HeLa cells cultured in the control medium for 0 to 4 days. Scale bars, 20 µm. (b) Intensity profiles of mitochondrial PT signals corresponding to the dotted lines in (a). The dashed-dotted lines represent the average peak values. (c) Temporal changes in the mitochondrial PT signal evaluated from the standard deviation (SD) of the pixel values in the PT images.

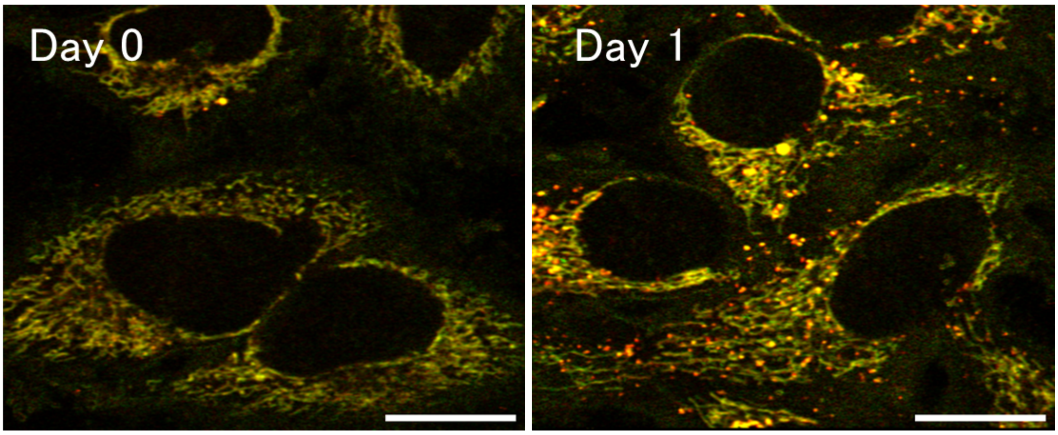

Fig. S3. Photothermal images of cells cultured in 2-DG-supplemented medium for 0 and 1 days. Scale bars, 20 μm.

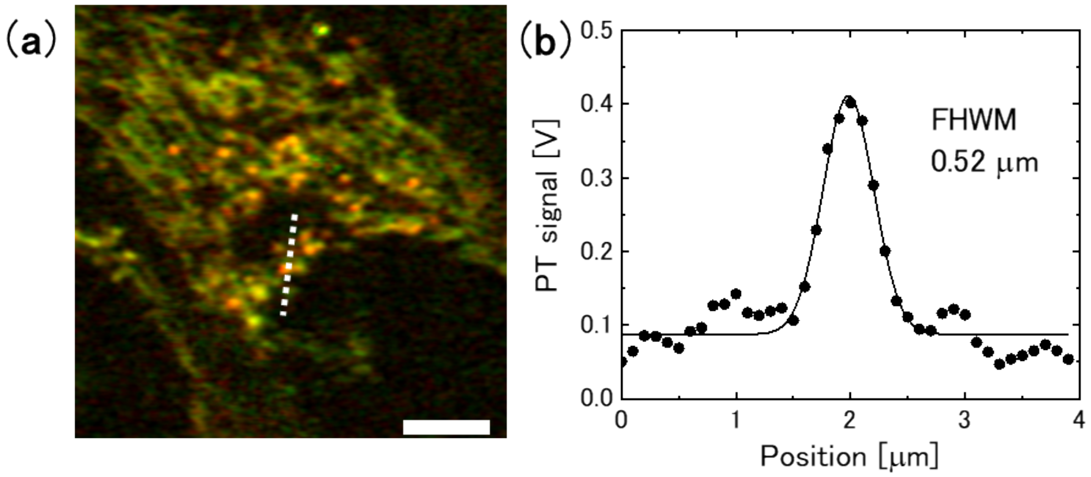

Fig. S4. Evaluation of lysosome size in a cell cultured in control medium. (a) Photothermal (PT) image of a part of the cell and (b) intensity distribution of a lysosome for the PT signal at 640 nm. Lysosome size was evaluated on the basis of the full width at half maximum (FWHM). The solid line indicates the fitting curve using a Gaussian function. Scale bar, 5 μm.

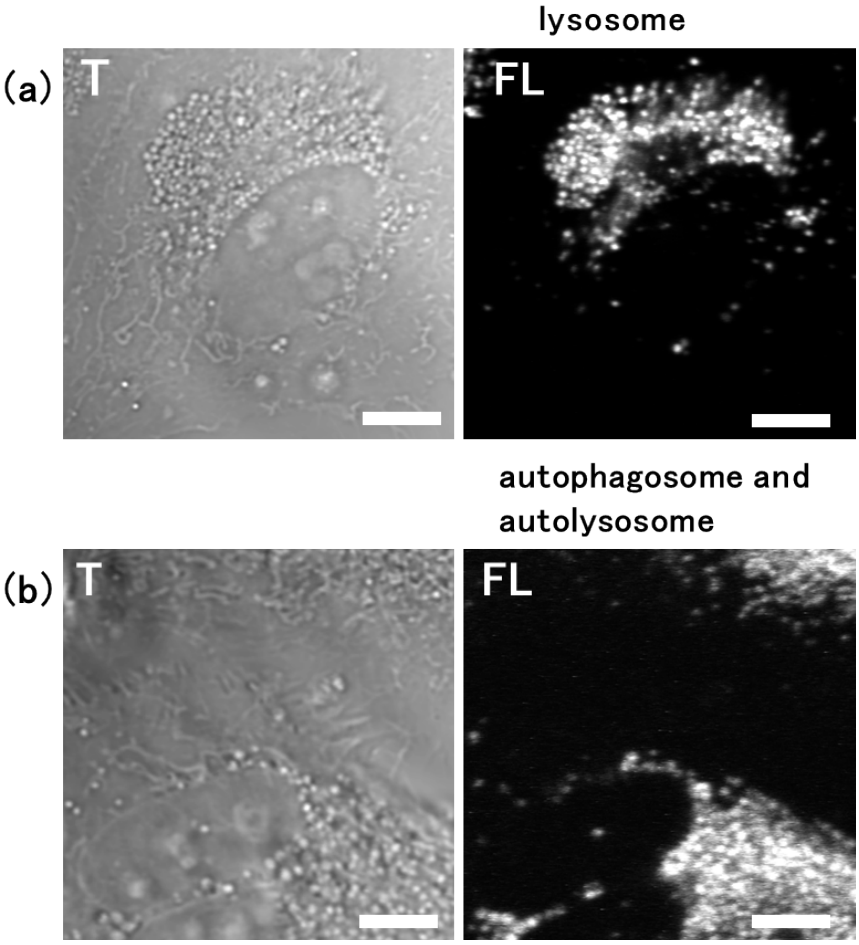

Fig. S5 Accumulation of hypertrophic lysosomes in a cell cultured in glucose-free medium. Hypertrophic lysosomes were identified using transmission (T) and fluorescence (FL) imaging. The cells were stained with (a) LysoPrime and (b) DAPGreen after 4 days of incubation in the glucose-free medium. For the fluorescence labelling of lysosomes, cells in the glass-bottom dishes were washed with Hanks' balanced salt solution (HBSS) and then stained with LysoPrime Deep Red (Dojindo) diluted 1,000-fold with HBSS for 30 min. Subsequently, the cells were washed with HBSS, after which imaging was conducted. Fluorescence images were acquired with an excitation wavelength of 640 nm. The labelling of autophagosomes and autolysosomes with DAPGreen was performed in a manner consistent with that described for Fig. 3. The punctate structures observed in the transmitted images exhibited co-localisation with the signals present in each fluorescent image, indicating that they are lysosomes (autolysosomes). Scale bars, 10 μm.

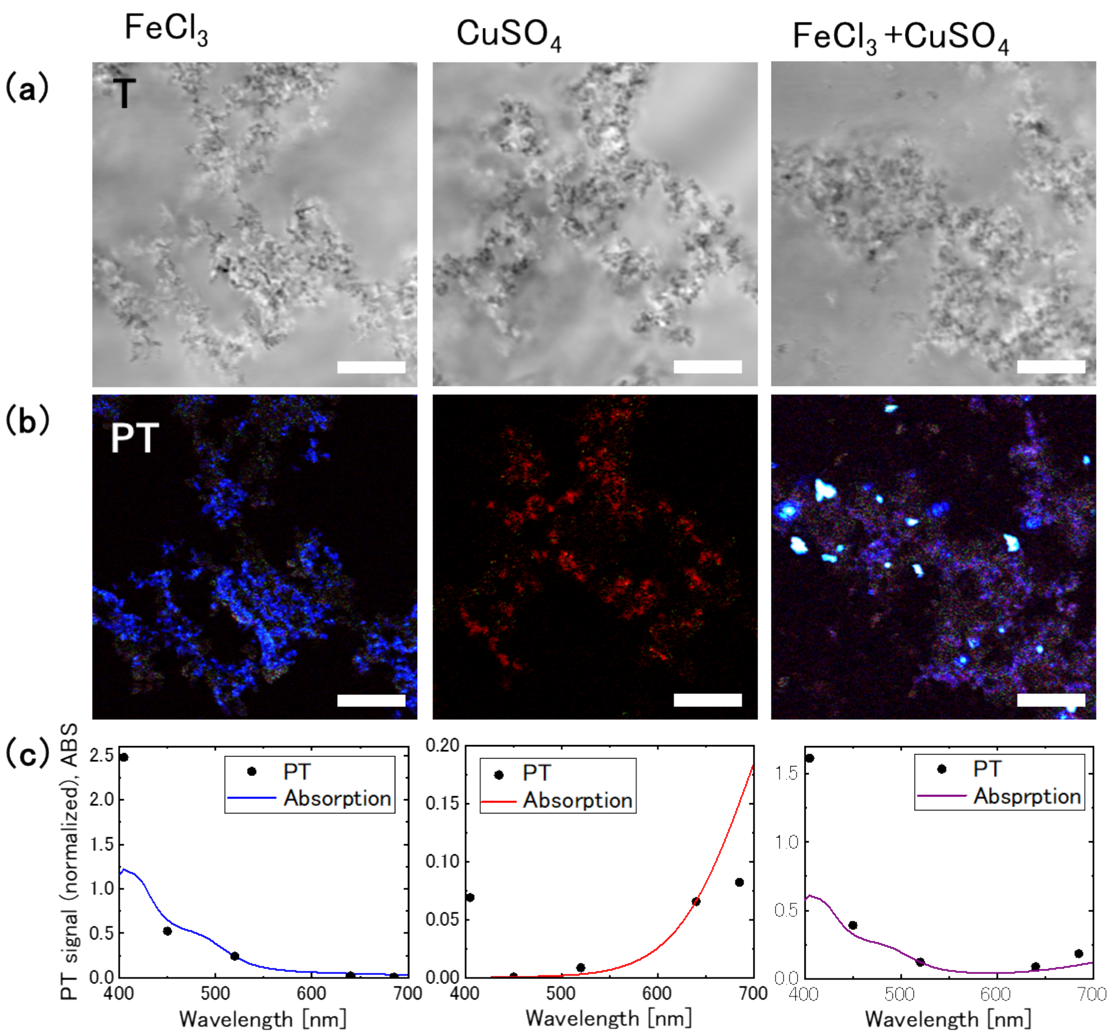

Fig. S6. (a) Transmission (T) and (b) photothermal (PT) imaging for albumin aggregates, the formation of which was induced by adding iron and copper ions or both. A solution containing 1% iron(III) chloride hexahydrate and 1% copper(II) sulphate was added to the 2% albumin solution at volume ratios of 1:100 and 1:200, respectively, to induce aggregation. A solution containing 0.5% iron(III) chloride hexahydrate and copper(II) sulphate was mixed with the albumin solution at a volume ratio of 1:100. The mixture containing the aggregates was vortexed vigorously and diluted to ~1/10th of the concentration using distilled water. Subsequently, 10 μL of the aqueous dispersion of the aggregates was dropped onto a glass slide and sealed with a coverslip. The RGB colour PT images were obtained by combining the three images obtained at pumping wavelengths of 640, 520, and 450 nm. The pump power incident on the sample was 1.9 mW at 405 nm and 3.6 mW at 450, 520 640, and 685 nm. The probe beam power was set to 11 mW. Scale bars, 20 μm. (c) Wavelength dependence of the PT signals obtained for the aggregates at the four wavelengths. The solid lines represent the absorption spectra of solutions containing ferric chloride hexahydrate, copper sulphate, and both these chemicals. The PT signals were normalised with respect to the absorbance of the metal ion solution at 640 nm. Aggregates containing iron appear blue in the combined RGB PT image because the iron ions have a higher absorption at short wavelengths. In contrast, aggregates containing copper ions appear red due to a higher absorbance at longer wavelengths.

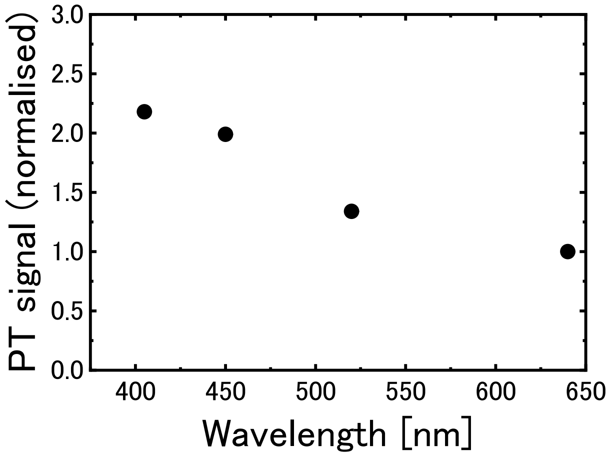

Fig. S7. Wavelength dependence of the PT signals for the aggresome-like structure. The signals are normalised with respect to that at 640 nm.

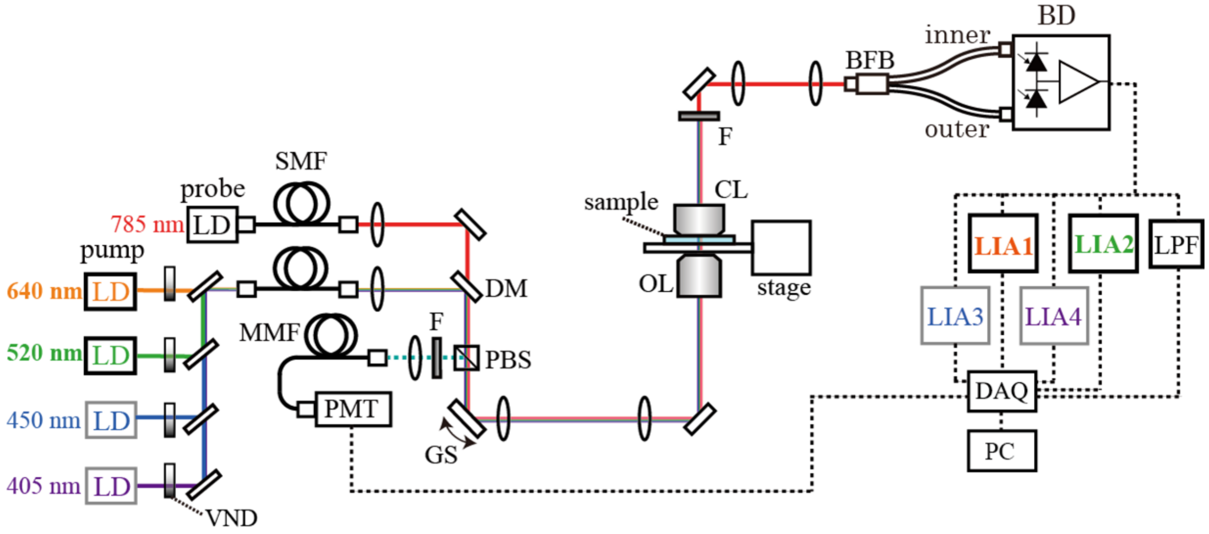

Fig. S8 Schematic diagram of the experimental setup used for four-wavelength PT imaging. LD: laser diode; VND: variable neutral density filter; SMF: single-mode fibre; DM: dichroic mirror; PBS: polarizing beam splitter; GS: galvano scanner; OJ: objective lens; CL: collection lens; F: filter; BFB: bifurcated fibre bundle; BD: balanced detector; LIA: lock-in amplifier; LPF: low-path filter with cut-off at 20 kHz.
